# Synthesis of Daidzein and Thiophene Containing Benzoxazine Resin and Its Thermoset and Carbon Material

**DOI:** 10.3390/molecules28135077

**Published:** 2023-06-28

**Authors:** Zhenhao Yao, Yin Lu, Jianan Song, Kan Zhang

**Affiliations:** Research School of Polymeric Materials, School of Materials Science and Engineering, Jiangsu University, Zhenjiang 212013, China; yaozhenhao89@gmail.com (Z.Y.); luy@stmail.ujs.edu.cn (Y.L.); songjianan@ujs.edu.cn (J.S.)

**Keywords:** benzoxazine, daidzein, thiophene, CO_2_ absorption, thermal stability

## Abstract

In this work, a novel bio-based high-performance bisbenzoxazine resin was synthesized from daidzein, 2-thiophenemethylamine and paraformaldehyde. The chemical structure was confirmed using nuclear magnetic resonance spectroscopy (NMR) and Fourier-transform infrared spectroscopy (FT-IR). The polymerization process was systematically studied using differential scanning calorimetry (DSC) and in situ FT-IR spectra. It can be polymerized through multiple polymerization behaviors under the synergistic reaction of thiophene rings with benzopyrone rather than a single polymerization mechanism of traditional benzoxazines, as reported. In addition, thermogravimetric analysis (TGA) and a microscale combustion calorimeter (MCC) were used to study the thermal stability and flame retardancy of the resulting polybenzoxazine. The thermosetting material showed a high carbon residue rate of 62.8% and a low heat release capacity (HRC) value of 33 J/gK without adding any flame retardants. Based on its outstanding capability of carbon formation, this newly obtained benzoxazine resin was carbonized and activated to obtain a porous carbon material doped with both sulfur and nitrogen. The CO_2_ absorption of the carbon material at 0 °C and 25 °C at 1 bar was 3.64 mmol/g and 3.26 mmol/g, respectively. The above excellent comprehensive properties prove its potential applications in many advanced fields.

## 1. Introduction

In recent years, the requirements for the comprehensive properties of materials with characteristic properties have become more and more stringent in various technical fields. Traditional materials often limit their performance due to structural limitations and cannot meet the requirements of today’s environment. High-performance polymers have attracted specific attention owing to their excellent thermal stability, light weight, good mechanical properties, great potential application prospect and processability in the fields of aerospace, the automobile industry, medical apparatuses and instruments, electronic packaging as well as coating adhesives [1]. They have also been gradually used deeper in the energy field, such as in wind blades, photovoltaic devices, the petroleum industry and fuel cells. Among many polymer materials, benzoxazine is a relatively new type of thermosetting resin with six-membered heterocyclic rings containing N and O atoms. Although benzoxazines have been in the public domain since the 1940s, it was not until the past 30 years that researchers have made substantial progress in the study of benzoxazine resins. It has attracted interest from academic researchers due to its high glass transition temperature (*T*g), near-zero shrinkage during curing, excellent chemical resistance, low coefficient of thermal expansion, flame-retardant properties, low dielectric constant and surface energy, low dielectric constant, good thermal stability, high mechanical properties and enormous molecular design flexibility [2,3,4]. On account of the advantages mentioned above, benzoxazine has gradually become a good substitute for many traditional thermosetting resins.

Polybenzoxazine thermoset with a three-dimensional network structure similar to phenolic resin was synthesized using cationic ring-opening polymerization of benzoxazine monomer. The typical polymerization temperature for benzoxazine is about 240 °C, and any catalysts and curing agents are not required during the whole polymerization process. The deformed chair conformation of the oxazine ring in the structure of the benzoxazine ring can be opened and then polymerized under certain external conditions. However, some shortcomings of traditional benzoxazines, such as high curing temperature, long polymerization time and brittleness, seriously limit the application of polymers [5,6]. In general, phenol, primary amine and formaldehyde are the typical raw materials for the synthesis of benzoxazines. According to the different reaction medium, the synthesis methods of benzoxazine monomer are also different, such as solution method, solve-free method as well as suspension method. Consequently, benzoxazine monomers with different structures can be obtained based on the utilization of various phenol and amine sources. In order to improve its performance, it is a common strategy to synthesize multifunctional benzoxazine monomers using novel structural designing. Monofunctional benzoxazine monomers prepared from phenols with monofunctional groups usually yield linear structures with an average molecular weight number of 500–2000 after thermal activation polymerization, and the resulting polymer has a low crosslink density. However, the thermal stability of bifunctional benzoxazines can be significantly improved by increasing the number of oxazine rings and crosslinking sites. Most of the reported studies on the synthesis and properties of polybenzoxazines have focused on bifunctional benzoxazine resins synthesized from bisphenol compounds. There is a usual method of synthesizing main-chain type benzoxazine using bisphenol resources such as bisphenol A [7], bisphenol F [8], bisphenol S [9], dihydroxynaphthalene [10] and so on. In addition, various functional groups such as acetylene [11], allyl [12], maleimide [13], norbornene [14] and nitrile [15] groups have been introduced into the molecular structure. And it is also possible to restrict the movement of molecular chains by introducing aromatic heterocyclic structures, significantly improving heat resistance and mechanical properties. Using molecular design to regulate the chemical reaction of the benzoxazine resin system can change the crosslinking and hydrogen bonding of the polymerization system and further promote the high performance of benzoxazine resin.

With the development of science and technology, the abuse of oil resources leads to the aggravation of global warming and other environmental problems. The proposal of the concept of “carbon peaking and carbon neutrality” has garnered heightened interest and scrutiny towards the spheres of environmental conservation and energy utilization. As a result, carbon capture and storage has been extensively studied. Adsorption technology has been developed efficiently in CO_2_ capture because of its advantages of simple technology, wide range of temperature and low energy demand [16,17,18]. Although the use of amine water solution to absorb CO_2_ has reached the commercial standard, the shortcomings of high energy consumption and equipment corrosion still need to be overcome [19]. Porous materials possess the ability to adsorb CO_2_ in an expeditious and reversible manner with minimal energy consumption, and have emerged as a promising solution. Porous carbon materials are a notable candidate in this regard, owing to their exceptional features such as high surface area, commendable chemical resistance and remarkable thermal stability towards acidic and alkaline media. Moreover, they are well-suited for CO_2_ capture in a diverse array of conditions [18]. However, under common environmental conditions, most activated carbons show an adsorbable capability less than 3 mmol/g of CO_2_ [20]. It has been found that porous carbon materials containing nitrogen and sulfur elements can interact effectively with acidic CO_2_ gas [21]. In order to improve the adsorption capacity, some carbon materials with N [22], S [23] and other atoms have been designed.

The use of biological-based phenol or amine sources to replace petroleum-derived raw materials to synthesize polymers has attracted increased attention [24,25,26]. In recent years, a large number of bio-based benzoxazines have been prepared from sustainable resources such as furfurylamine [27,28], glycine [29,30], cardnol [31], guaiacol [32,33], coumarin [34,35], phloretic acid [36] and dehydroabietylamine [37,38]. Soybean has a high yield among all crops, and it has been found that as much as 41.7% daidzein is involved in soy germ. Daidzein (7-hydroxyl-3(4-hydroxylphenyl)-4H-chromen-4-one) has found widespread utilization in the realms of food and medicine due to its inherent bioactive properties such as antioxidant, antibacterial and anti-inflammatory effects [39]. Moreover, daidzein contains two phenolic hydroxyl groups, and has high designability as a phenol source for benzoxazine synthesis. In addition to the phenolic hydroxyl group, the special structure of benzopyrone can improve the activity of polymerization. Recent studies have found that both benzoxazine and epoxy resins synthesized from daidzein have very high thermal stability and excellent flame-retardant properties [40,41]. On the other hand, the thiophene ring is a kind of five-membered heterocyclic rings containing S atoms, which have high reactivity and may play a role in crosslinking point during polymerization reactions [42].

Based on the above inspiration, we successfully prepared a difunctional benzoxazine using daidzein and 2-thiophenemethylamine as starting materials. The chemical structure of benzoxazine was characterized using nuclear magnetic resonance (NMR) and Fourier infrared (FT-IR) spectroscopies. Moreover, the polymerization behavior of benzoxazine was investigated using differential scanning calorimetry (DSC) and in situ FT-IR spectroscopy; we also analyzed the activation energy of it polymerization. In addition, the thermal stability and flame retardancy of cured benzoxazine as well as the CO_2_ absorption of its corresponding carbon material was evaluated. It provides a sample for the application of a bio-based benzoxazine system in the field of carbon materials.

## 2. Results and Discussion

### 2.1. Synthesis of the Benzoxazine Monomer

**Scheme 1:**
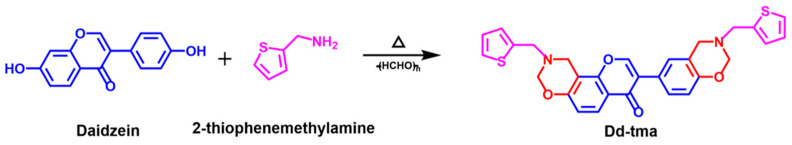
Synthesis of benzoxazine based on daidzein and 2-thiophenemethanamine.

In this work, a new precursor benzoxazine with a thiophene functional group was prepared via a multi-component one-pot reaction using daidzein and 2-thiophenemethyla-mine as the carbon and nitrogen sources. The synthetic reaction mechanism of benzoxazine is shown in Scheme 1. Benzoxazine monomer was first synthesized via a Mannich condensation reaction of 2-thiophenemethylamine, daidzein and paraformaldehyde without any catalyst. Then, benzoxazine was opened to form oxazine rings at a temperature of 140–240 °C to generate a three-dimensional network of a crosslinked structure.

**Figure 1:**
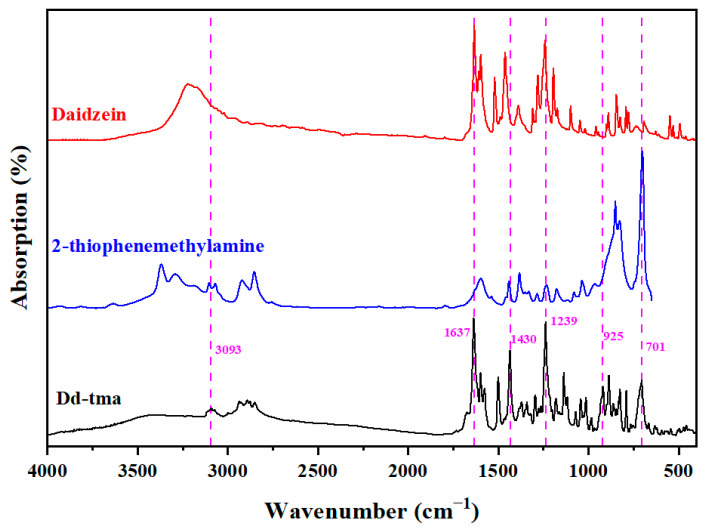
FT-IR spectrum of **Dd-tma**, daidzein and 2-thiophenemethylamine.

Scheme 1 outlines the triumphant synthesis of **Dd-tma**, initiated with the utilization of raw materials comprising daidzein, 2-thiophenemethylamine and paraformaldehyde. The chemical structure of **Dd-tma** was characterized using ^1^H and ^13^C NMR and FT-IR spectra. Figure 1 shows the FT-IR spectrum of **Dd-tma**, daidzein and 2-thiophenemethyl-amine, in which the typical bands indicate that our newly designed benzoxazine contains daidzein, an oxazine ring and thiophene groups [43]. Moreover, The infrared spectra of **Dd-tma** showed typical absorption peaks on thiophene: 3093 (in-plane bending vibration of C-H), 1430 (symmetric stretching vibration of C=C) and 701 (antisymmetric stretching vibration of C-S) [44]. The sharp signal shown at 1639 cm^−1^ was assigned to the C=C-C=O group in the daidzein units [45]. The characteristic signal at 1237 cm^−1^ was due to the asymmetric stretching vibration of the C-O-C group in the oxazine ring; it was also observed in the spectrum of daidzein [14]. In addition, the characteristic band of the correlation pattern of oxazine rings was located at 925 cm^−1^ [46].

**Figure 2:**
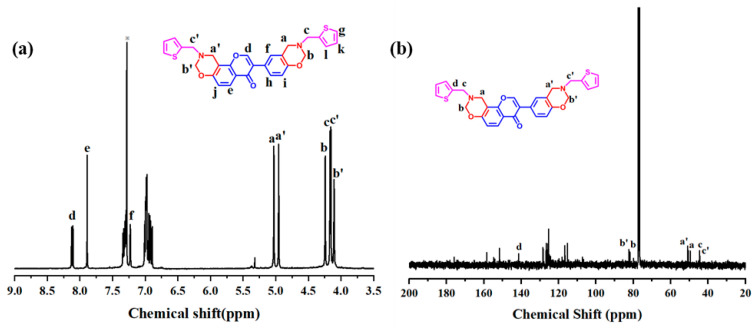
^1^H (**a**) and ^13^C (**b**) NMR spectra of **Dd-tma** (lowcase in (**a**): Hydrogen proton, lowcase in (**b**): Carbon atom).

In order to obtain more detailed structure information, the benzoxazine monomer was also analyzed using ^1^H and ^13^C NMR. As shown in Figure 2a, two set of signals at 4.96 and 5.03 ppm and 4.11 and 4.24 ppm were used for **Dd-tma**, which can be assigned to N-C*H*_2_-O and Ar-C*H*_2_-N in oxazine rings, respectively. The repetition of these peaks corresponding to oxazine rings was caused by the asymmetric chemical structure of daidzein [47]. Chemical shifts of two methylene groups in 2-thiophenmethylamine were observed at 4.16 and 4.17 ppm as two distinct resonances. The formants in the 6.9–7.3 ppm range were attributed to the interaction of thiophene rings with aromatic protons. In addition, the integral ratio of H_b_ to H_d_ was 4:1, indicating that a bis-benzoxazine monomer was formed in the current study. Other detailed proton assignments can be found in Section 3.

Figure 2b shows the ^13^C NMR spectrum of **Dd-tma**. A pair of signals at 50.95 and 44.48 ppm corresponded to the Ar-*C*H_2_-N- group, while the characteristic peaks at 81.82 and 82.35 ppm corresponded to the -O-*C*H_2_-N- group of the oxazine ring. The characteristic signals at 50.43 and 49.61 ppm were assigned to the two methylene carbon resonances of the 2-thiophenemethylamine portion. The above results based on FT-IR and NMR spectra clearly indicate that **Dd-tma** was successfully synthesized.

### 2.2. Polymerization Behaviors and Kinetics of Dd-tma

**Figure 3:**
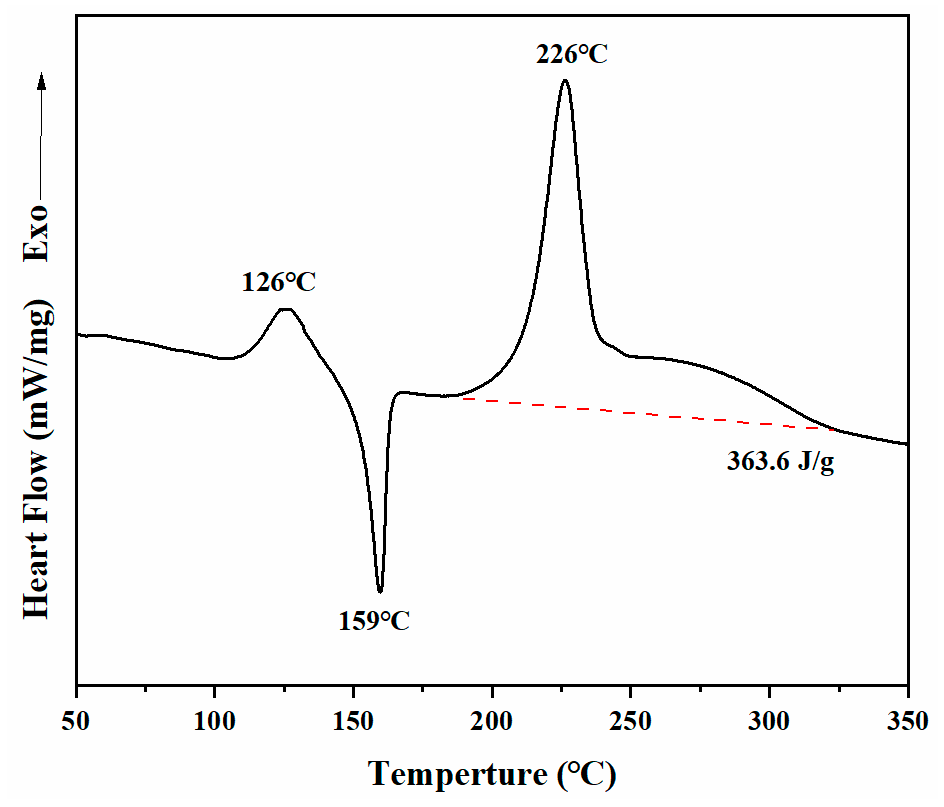
DSC thermograms of **Dd-tma**.

The thermal behaviors of **Dd-tma** were investigated with both DSC and in situ FT-IR analyses. The purity of the benzoxazine may lead to different DSC images of the same group of benzoxazine samples. Therefore, we used only very pure samples of benzoxazine monomer in our current study, which would demonstrate a reliable mechanism for thermo-activated ring-opening polymerization of benzoxazine. As shown in Figure 3, there was a sharp endothermic peak at 159 °C, which was caused by the melting of **Dd-tma**. Such a sharp melting peak also indicates its excellent purity. The initial temperature of ring-opening polymerization was about 190 °C, and the peak value of exothermic polymerization was observed at 226 °C. Moreover, the enthalpy value of polymerization was 363.6 J/g. Generally, the typical polymerization temperature of the oxazine ring was about 220–260 °C [48]. Such a relatively low polymerization temperature of **Dd-tma** may be the result of the involvement of the thiophene ring in the crosslinking reaction [42]. In addition, due to the regular structure and high purity, crystallization of the monomer could occur, which appeared as a small exothermic peak with a peak temperature of 126 °C prior to melting behavior.

**Figure 4:**
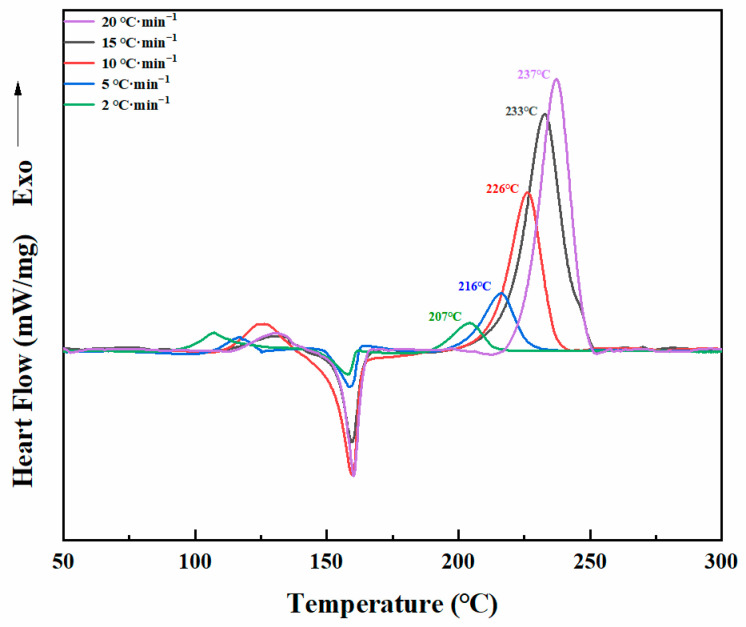
DSC curves of **Dd-tma** at different heating rates.

The activation energy (*E_a_*) of the daidzein-based benzoxazine monomer during the curing process was studied using the non-isothermal method by DSC at heating rates of 2, 5, 10, 15 and 20 °C/min, respectively, as shown in Figure 4. The *E_a_* of polymerization was calculated using the Kissinger and Ozawa theory [49,50]. The Kissinger equation, Equation (1), is as follows:

(1) $$ln(\frac{\beta }{T_{p}^{2}})=ln(\frac{AR}{E_{a}})-\frac{E_{a}}{RT_{P}}$$

**Figure 5:**
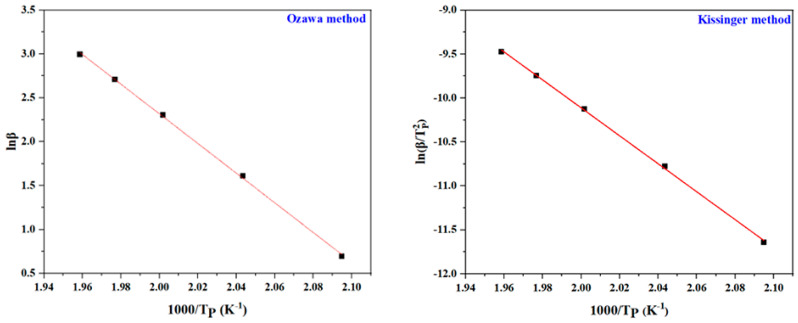
Representations of Kissinger and Ozawa methods for calculation of Ea values for **Dd-tma**.

In addition, the modified Ozawa method, Equation (2), was applied as follows:

(2) $$ln\;\beta =-1.052\frac{E_{a}}{RT_{P}}+C$$

where the heating rate is represented by β, A is the pre-exponential factor, the peak heat release temperature is *T_P_*, the gas constant is *R* and the constant is *C*. Two fitted straight lines were obtained, respectively, in the corresponding figure according to the corresponding methods (Figure 5). The *E*_a_ values based on the Kissinger and Ozawa methods were calculated to be 125.3 kJ/mol and 133.48 kJ/mol. The calculated *E_a_* value of **Dd-tma** polymerization was lower than many reported bifunctory benzoxazine monomers [14,51,52]. The result might be due to the cooperative activation effect of benzopyrone and thiophene [42].

**Figure 6:**
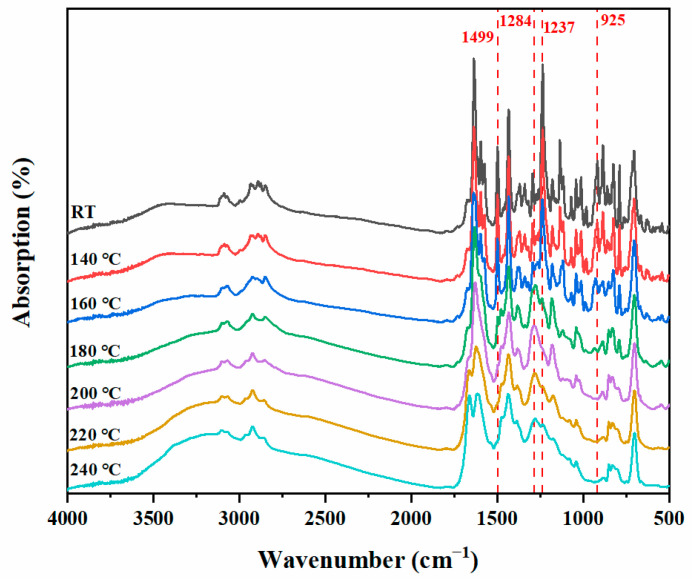
In situ FT-IR spectra of **Dd-tma** during the step-by-step polymerization approach.

**Scheme 2:**
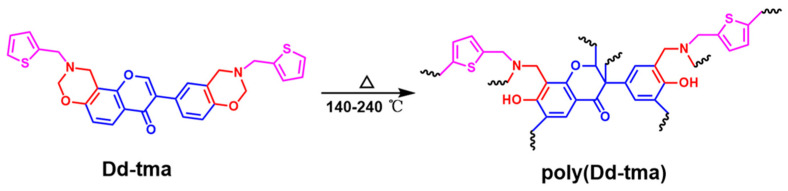
Proposed polymerization behaviors of **Dd-tma**.

In situ FT-IR analysis was used to study the polymerization behavior of benzoxazine. As shown in Figure 6, the characteristic band at 1237 cm^−1^ and the related mode characteristic band at 925 cm^−1^ of the oxazine ring caused by asymmetric stretching vibration of the C-O-C group in the oxazine ring of poly(**Dd-tma**) gradually disappeared during the heating process. Meanwhile, the broad bands gradually appeared in the range of 3600–3000 cm^−1^, which can be attributed to multiple stretching patterns of phenolic hydroxyl groups emerging as the temperature increased [53]. In addition, the characteristic olefin band at 1499 cm^−1^ decreased and the band at 1284 cm^−1^ increased, suggesting the further polymerization of the C=C band in the daidzein structure of **Dd-tma**. Meanwhile, the typical band of C-H stretching on the thiophene ring at 885 cm^−1^ and 3088 cm^−1^ decreased with the increasing temperature. The characteristic bands of Schiff bases (-N=CH-) in the range 1610–1670 cm^−1^ were due to the formation of a polymeric intermediate via ring opening of the oxazine ring. Therefore, we propose the thermal activation polymerization process of poly(**Dd-tma**) based on the results obtained using DSC and in situ FT-IR analysis (Scheme 2).

### 2.3. Thermal Properties of Poly(Dd-tma)

**Figure 7:**
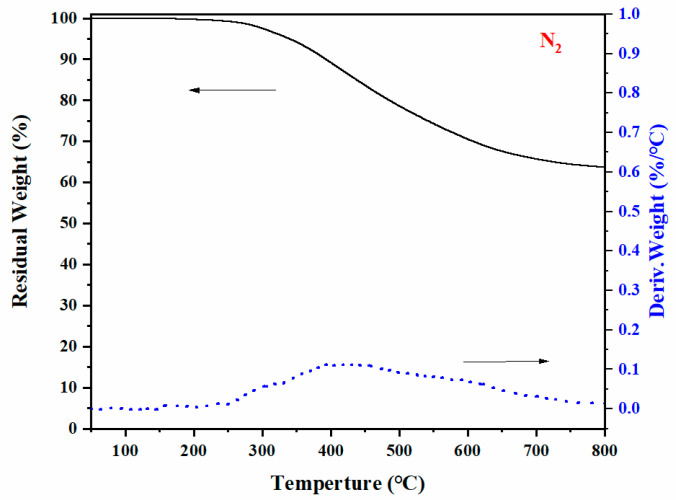
Thermogravimetric analysis of poly(**Dd-tma**) in nitrogen.

The thermal stability of poly(**Dd-tma**) was studied using TGA in a nitrogen atmosphere, as seen in Figure 7. poly(**Dd-tma**) showed T_d5_ (5 % weight loss temperature) and T_d10_ (10% weight loss temperature) values of 326 °C and 384 °C, respectively. Moreover, the TGA curve showed a high char yield (Yc) value of 62.8% at 800 °C in nitrogen. Compared with the traditional benzoxazine resin, the thermal stability of polybenzoxazine was greatly improved due to the special benzopyrone structure of daidzein and the presence of thiophene. The wide DTG peak indicates that the decomposition rate of resin was very slow in a wide temperature range. The low rate of decomposition during thermal degradation is very important from an ignitability point of view. Recent reports have shown thermosetting resins derived from thiophenyl benzoxazine usually have good thermal stability [42,44,54]. Then, we roughly evaluated the flame-retardant capability using the limiting oxygen index (LOI), which was calculated by applying the van Krevelen equation by the Yc value in a nitrogen atmosphere [55]:

LOI% = 17.5 + 0.4(Yc)(3)

The calculated LOI value of Dd-tma at 800 °C was as high as 44.6%, and the polymers with an LOI value greater than 28% were considered to be self-extinguishing [56]. The obtained LOI value indicates that it had excellent flame retardancy.

**Figure 8:**
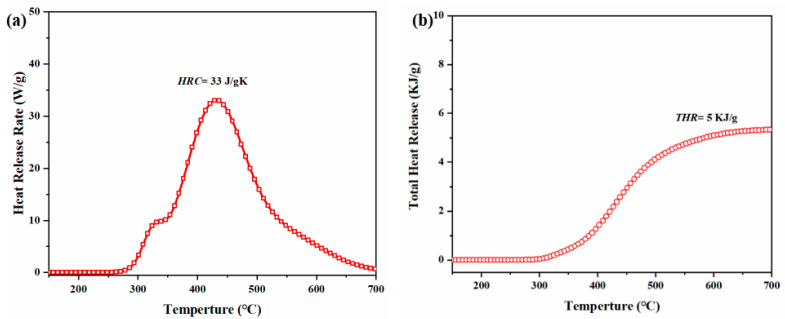
Heat release rate (**a**) and total heat release (**b**) vs. temperature for poly(**Dd-tma**).

The flammability of poly(Dd-tma) was evaluated quantitatively using microscale combustion calorimetry (MCC). Figure 8a shows the heat release rate (HRR) as a function of temperature, where the HRR maximums were observed at 310 °C. The value of heat release capacity (HRC) can be calculated according to the information in Figure 8b, which was 33 J/gK. Additionally, the total heat release (THR) value of poly(**Dd-tma**) was 5 kJ/g. The HRC value of poly(**Dd-tma**) obtained in this study was much lower than that of 47 polymers previously reported [57]. It is known that materials with an HRC of less than 300 J/gK are considered as self-extinguishing, while ones with an HRC of less than 100 J/gK are considered as non-combustible materials [58]. The HRC value was greatly reduced due to the nitrogen and sulfur in the monomer and the highly crosslinked network structure. These results indicate that benzoxazine prepared from daidzein and 2-thiophenemethylamine possesses good flame retardancy. Therefore, the results of this study demonstrate the potential utilization of this polybenzoxazine in fire protection applications.

### 2.4. CO_2_ Capture Capacity of Porous Materials

**Figure 9:**
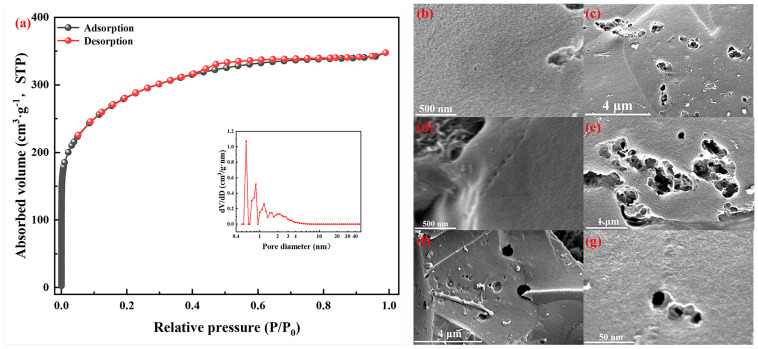
(**a**) Nitrogen adsorption–desorption isotherms at −196 °C and pore size distribution (PSD) and (**b**–**g**) SEM images.

**Figure 10:**
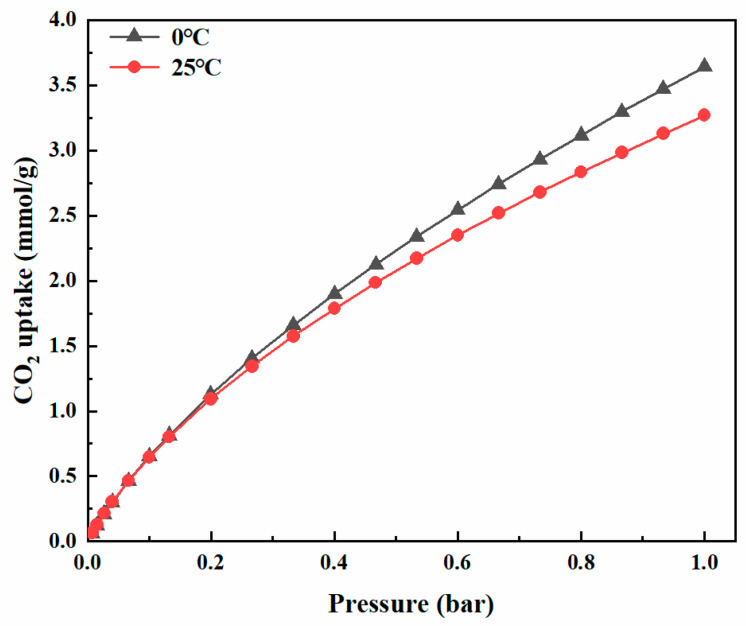
CO_2_ adsorption capacity at 0 °C and 25 °C.

Figure 9a shows the N_2_ adsorption–desorption curve and pore size distribution of porous carbon material at −196 °C. It is well-known that KOH is usually used as a chemical activator to prepare microporous carbon materials [59]. After activation, nitrogen uptake was significant when P/P_0_ was less than 0.05, indicating the presence of a large number of micropores. According to IUPAC classification, the adsorption–desorption isotherm of carbon material was a typical type I [60]. The presence of hysteresis loops in the figure indicates the presence of mesoporous pores [61], and the PSD curve in the figure also shows the presence of mesoporous pores in the range of 2.0–3.8 nm. The BET surface area of carbon material was 917 m^2^/g, the total pore volume was 0.53 cm^3^/g and the average pore size was 2.59 nm. It can be seen from the PSD diagram that there were abundant micropores between 0.5 and 2.0 nm. The pore size of the material was mainly concentrated at 0.5 nm, 0.8 nm, 1.1 nm and 1.5 nm. It had a relatively narrow aperture distribution. It had a relatively wide aperture distribution at 2–3.8 nm. As shown in Figure 9b–g, a small number of large pores existed in carbon material, which may be attributed to the release of residual solvent bubbles in the curing process and the structural damage of pore walls etched by the activation agent during KOH activation, such as the fracture of pore walls between adjacent pores [10]. It can be seen in Figure 9b that there were abundant pothole-like micropores on the surface of the material, and it can also be seen in Figure 9d that there were continuous micropores. Carbon materials were finally used for CO_2_ capture, and the adsorption isotherm measurement is shown in Figure 10. Due to the high BET surface area and the presence of basic N and S atoms, the CO_2_ absorption amounts reached 3.64 mmol/g and 3.26 mmol/g at 0 °C and 25 °C at 1 bar, respectively. These values are comparable to those recorded from other carbon materials [10,21,62].

## 3. Materials and Methods

### 3.1. Materials

Daidzein, 2-thiophenemethylamine, paraformaldehyde and F127 were obtained from Sigma-Aldrich. Hexane, xylene, ethyl acetate, Potassium hydroxide(KOH) and sodium hydroxide (NaOH) were purchased from Aladdin Reagent, China and used as received.

### 3.2. Characterization

^1^H and ^13^C NMR spectra were recorded using a Bruker AVANCE (400 MHz) in deuterated chloroform at room temperature using tetramethylsilane as internal standard. The FT-IR spectrum was obtained using a Nicolet AVATAR-360 spectrophotometer and deuterated sulfate tripeptide detector with a resolution of 4 cm^−1^. The samples were ground using potassium bromide (KBr) and pressed into KBr discs for testing. Differential scanning calorimetry (DSC) thermograms were obtained with a NETZSCH 204f1 instrument under a nitrogen atmosphere at a temperature rise rate of 10 °C/min. Different heating rates were also measured to calculate the activation energy (*E_a_*) value. Thermogravimetric analysis (TGA) was performed with a NETZSCH STA449-C thermogravimetric analyzer for the thermal stability from room temperature to 800 °C at a heating rate of 10 °C/min under N_2_. N_2_ adsorption was performed using a Micromeritics ASAP 2460 adsorption analyzer at −196 °C. Before measurement, the sample was vacuum treated at 100 °C for 6 h. The specific surface area and pore structure were obtained with the BET method. CO_2_ adsorption isotherms were measured at 0 °C and 25 °C using a Micromeritics TriStar II 3020 analyzer. A microscale combustion calorimeter (MCC, FAA-PCFC) was used to study the flammability of polybenzoxazine. A heating rate of 1 K/s and an 80 mL/min stream of nitrogen were applied for the measurement from room temperature to 750 °C. In addition, the anaerobic pyrogen decomposition products in N_2_ flow were mixed with an O_2_ flow of 20 mL/min and sent to the combustion furnace (the temperature was set at 900 °C).

### 3.3. Synthesis of 9-(theiophen-2-ylmethyl)-3-(3-(thiophen-2-ylmethyl)-3,4-dihydro-2H-benzo[e]-[1,3]oxazin-6-yl)-9,10-dihydro-4H,8H-chromeno[8,7-e][1,3]oxazin-4-one (Abbreviated as Dd-tma)

Xylene was used as the solvent for this reaction. Daidzein (1.00 g, 0.0039 mol), paraformaldehyde (0.52 g,0.0173 mol), 2-thiophenemethylamine (0.89 g, 0.0079 mol) and 50 mL of xylene were added into a 100 mL round-bottom flask equipped with a condenser. The chemical mixture was stirred by a magnetic stirrer. The reaction temperature rose from RT to 130 °C within 5 min and remained at 130 °C for 9 h. After cooling to room temperature, the obtained solution was washed three times with 1 N NaOH aqueous solution and three times with distilled water. The organic solvent was removed using a rotating evaporator and purified via column chromatography with a hexane and ethyl acetate mixture (4:1) (yield ca.51%). ^1^H NMR (400 MHz, CDCl_3_), ppm: *δ* = 8.12 (d, 1H, H_d_), 7.89 (s, 1H, H_e_), 7.32 (m, 3H, H_g_ and H_h_), 7.23 (d, 1H, H_f_), 7,01 (m, 1H, H_i_), 6.95 (m, 5H, H_l_, H_k_ and H_j_), 5.03 (s, 2H, H_a_), 4.96 (s, 2H, H_a’_), 4.24 (s, 2H, H_b_), 4.16 (m, 4H, H_c_ and H_c’_), 4.11 (s, 2H, H _b’_). FT-IR spectra (KBr), cm^−1^: 1639 (C=O stretching), 1430 (C=C stretching of thiophene ring), 1237 (C-O-C antisymmetric stretching), 925 (benzoxazine-related band).

### 3.4. Polymerization of Dd-tma

The benzoxazine monomer **Dd-tma** was polymerized in a stainless steel mold by carrying out multiple polymerizations steps of 1 h each at the following temperatures, including 140, 160, 180, 200, 220 and 240 °C, respectively, therefore obtaining poly(**Dd-tma**).

### 3.5. Preparation of Porous Carbon Material

3 g **Dd-tma** and 1.5 g F127 were added into 50 mL ethanol. The whole mixture was stirred at 70 °C for 1 h and evaporated at room temperature for 24 h. The resulting mixture was heated step-by-step for 1 h in ovens at 140, 160, 180, 200, 220 and 240 °C, respectively. The cured material was heated for 5 h at 600 °C under a nitrogen atmosphere at the heating rate of 5 °C/min for further carbonization. Then, the sample obtained after carbonization was mixed with KOH aqueous solution (mass ratio of carbonized PBZ to KOH = 1:2), and then stirred at room temperature for 24 h. The water was evaporated after the stirring. The activation process was in a tubular furnace at 800 °C for 1 h under nitrogen flow with a heating rate of 5 °C/min. The product was washed and filtered with deionized water until the PH value of a filtrate that had reached neutral, and finally dried at 110 °C for 12 h to obtain porous carbons.

## 4. Conclusions

A new benzoxazine monomer, **Dd-tma**, was synthesized from daidzein, 2-thiophenemethylamine and paraformaldehyde in the current study. The structure of **Dd-tma** was characterized using NMR and FT-IR. In addition, the obtained polybenzoxazine possessed a T_d5_ of 326 °C, T_d10_ of 384 °C and Yc of 62.8%, indicating its good thermal stability. The thermosetting resin also showed self-catalytic and self-extinguishing properties without the addition of any catalyst or anti-combustion additives. Notably, poly(**Dd-tma**) exhibited both very low HRC (33 J/gK) and THR (5 KJ/g) values. Moreover, its carbon material exhibited high specific surface area and good CO_2_ capture capability. This study suggests that our newly synthesized benzoxazine has excellent prospects in the fields of porous material and flame-retardant materials.

## Data Availability

Data will be made available on request.

## Sample Availability

Samples of the benzoxazine monomer are available from the authors.
